# Territorial Disparities in Dental Care for Disabled Persons and Oral Health-Related Indicators: A Population-Level Approach in Brazil’s Public Healthcare System from 2014 to 2023

**DOI:** 10.3390/ijerph21050632

**Published:** 2024-05-16

**Authors:** Ricardo Barbosa Lima, Aluísio Eustáquio de Freitas Miranda-Filho, Ana Paula Gomes e Moura, Paulo Nelson-Filho, Léa Assed Bezerra da Silva, Raquel Assed Bezerra da Silva

**Affiliations:** 1 Graduate Program in Pediatric Dentistry, School of Dentistry of Ribeirão Preto, University of São Paulo, Ribeirão Preto 14040-904, SP, Brazil; 2 Department of Pediatric Dentistry, School of Dentistry of Ribeirão Preto, University of São Paulo, Ribeirão Preto 14040-904, SP, Brazil

**Keywords:** healthcare disparities, dental care, disabled persons, health services accessibility

## Abstract

This study evaluated territorial disparities in dental care for disabled persons in Brazil’s public healthcare system from 2014 to 2023. The person-year incidence of outpatient dental procedures carried out by special care dentistry specialists and hospitalizations for dental procedures for disabled persons were compared across different regions and against the national estimate. In addition, productivity was correlated with oral health-related indicators. The significance level was set at 5%. The northern region exhibited the highest outpatient productivity, while the southern region showed lower productivity compared to the national estimate (both *p*-value < 0.05). This pattern was reversed in inpatient productivity (both *p*-value < 0.05), with the northeastern and central–western regions also below average (both *p*-value < 0.05). There were no significant correlations between the indicators and inpatient productivity, but outpatient productivity was positively correlated with the proportions of inhabitants who self-rated their general and oral health as “poor” or “very poor”, who have never visited a dentist, and who visited a dentist for tooth extraction (all *p*-values < 0.05). Territorial disparities in dental care for disabled persons were observed within Brazil’s public healthcare system, and they were correlated with unfavorable oral health-related indicators at the population level.

## 1. Introduction

Access to oral healthcare services and the provision of appropriate dental care are essential for oral health, also including oral health-related quality of life (OHRQoL) [1]. In recent years, dental researchers worldwide have focused their efforts on understanding oral health and OHRQoL in various populations, territories, and contexts using different approaches. Indeed, the current studies addressing OHRQoL outcomes use specific instruments for this purpose, but also explore other oral health-related indicators, such as access to dental care and its impact on individuals from the perspective of oral health-related well-being as a multidimensional outcome [1,2]. Currently, there is no shortage of evidence corroborating the impact of oral health on general health (including general health-related quality of life, HRQoL), although diverse aspects of OHRQoL have not yet been extensively explored [2].

However, for disabled persons, accessing oral healthcare services appropriately and receiving adequate dental care can be challenging, highlighting concerns about the OHRQoL of this population. Although relevant, studies investigating OHRQoL aspects in disabled persons are scarce [3]. Thus, it is also important to note that individuals with disabilities may experience oral health illness in a different manner compared to those without a disability. Indeed, there is not one type of disability (e.g., physical, cognitive, mental, and other physiological impairments, such as comorbidities and medications). However, overall, one or more disabilities can directly impact oral health (and consequently OHRQoL), considering abnormalities in the oral cavity and associated structures, systemic changes that limit dental care in typical outpatient settings, or physical and cognitive impairments that reduce the ability for self-care, such as oral hygiene actions [3,4,5].

In addition, some physical and cognitive impairments can negatively influence other daily life activities, such as education and work, often contributing to socioeconomic vulnerability that is directly linked to OHRQoL [2,4,5]. Likewise, it is important to consider mental illness and its impact on access to dental care and OHRQoL, as demonstrated by previous studies, highlighting the negative impact of psychiatric comorbidities on the provision of effective dental treatments [6,7]. As a reflection of this panorama, it is not uncommon for disabled persons to experience a high frequency of untreated oral diseases and to require specialized dental care, such as inpatient assistance (hospitalizations) [3,4].

When connecting the relevance of dental care to the OHRQoL of disabled persons, it is known that the geographic location (where people live) is a determining factor in access to oral healthcare services and, consequently, in addressing oral health needs effectively [8,9]. Therefore, it is reasonable to understand why territorial disparities may contribute to OHRQoL inequities at some level [1]. The importance of geography for oral health can be observed when considering that the prevalence of oral diseases is higher in low- and middle-income countries, where the population often experiences health vulnerability due to poverty and social marginalization [8]. From the same perspective, using real-world data, a previous study demonstrated that in Brazil (a country with vast territorial dimensions and five distinct regions), 84.9% of caries-related hospitalizations were concentrated in one region (southeast), which was not proportional to its population or epidemiological demands. This outcome suggested a spatial concentration of dental care provided, revealing significant differences in access to oral health assistance between regions within the same country, and serves as a valuable example of geographic-related (territorial) disparities [9].

Structuring a rationale, all these perspectives and evidence raise a pivotal research question regarding OHRQoL: are there territorial disparities in dental care provided for disabled persons in Brazil? In response to this question, a positive answer would suggest that inequities are likely to be observed in the OHRQoL, hypothesizing the impact of insufficient access to oral health services and the persistence of unmet oral health demands, also considering unfavorable oral health-related indicators. Furthermore, it is also possible to question whether territorial disparities, if they exist, are correlated with the oral health-related indicators.

Moreover, this context underscores the impact of public oral healthcare services for disabled persons, as socioeconomic vulnerability can hinder access to oral healthcare through private means [4,9]. Brazil, for instance, has a nationwide public healthcare system in which dental care is part of the covered assistance. Public dental services are available at different levels of complexity (primary, secondary, and tertiary care—general and specialized assistance) within healthcare networks, with universal access. However, considering its vast territorial dimensions, oral health-related territorial disparities have been observed [9,10], which makes it essential to obtain an answer to the previously presented research questions.

To date, there has been no nationwide robust investigation addressing dental care for disabled persons from the perspective of territorial disparities, which is important for understanding OHRQoL in a multidimensional manner in this country. Therefore, the objective of this study was to evaluate territorial disparities in dental care for disabled persons in Brazil’s public healthcare system, exploring the correlation between dental care for disabled persons and oral health-related indicators through a population-level approach. The first null hypothesis (H0_1_) was that there is no territorial disparity in dental care for disabled persons, whether in outpatient or inpatient settings. The second null hypothesis (H0_2_) was that there is no correlation between productivity (annual quantity of outpatient dental procedures or hospitalizations) and oral health-related indicators.

## 2. Materials and Methods

### 2.1. Study Design and Ethics

A nationwide register-based study was designed using a time series approach, involving data available in open access. This study employed quantitative resources for longitudinal and retrospective analysis. All data used in the study were freely accessible in the public domain, allowing for a comprehensive evaluation of trends and patterns over time in a population-level approach, similar to other investigations [9,11]. Consequently, ethical approval was not required for this type of study. The methods did not involve any interaction, direct or indirect, with disabled persons or other human subjects assisted by Brazil’s public healthcare system. Instead, the analysis focused on aggregated data regarding the provision of outpatient and inpatient dental care within this public healthcare system. This approach aligns with Brazil’s ethical issues, particularly outlined in National Health Council—Ministry of Health Resolution 510, dated 7 April 2016 [12].

Delineating the approach, the territory of Brazil was stratified into two levels: regional (north, northeast, southeast, south, and midwest) and state-level (26 states and the Federal District). The first level was used to explore territorial disparities, while the second level was utilized to correlate productivity (outpatient and inpatient dental care for disabled persons) with oral health-related indicators. The timeframe was adjusted to the past ten years with annual data available for analysis (from 2014 to 2023). During this interval, all productivity from Brazil’s public health services was considered (without restrictions regarding the type of dental services).

### 2.2. Data Sources and Variables

The primary data sources regarding the productivity of Brazil’s public healthcare system were: (1) the National Outpatient Information System (*Sistema de Informações Ambulatoriais*—SIA/SUS) and (2) the National Hospital Information System (*Sistema de Informações Hospitalares*—SIH/SUS). Both information systems are provided by the Department of Informatics of Brazil’s public health system (DATASUS) [13].

From the SIA/SUS, the annual quantity of outpatient dental procedures carried out by special care dentistry specialists was retrieved, serving as a proxy measure for outpatient dental care for disabled persons. This quantity is registered monthly by the SIA/SUS based on the procedures recorded in the Outpatient Productivity Bulletins (*Boletins de Produtividade Ambulatorial*—BPAs) [11,13]. From the SIH/SUS, the annual quantity of hospitalizations for dental care of disabled persons was retrieved, serving as a proxy measure for inpatient dental care. Likewise, this quantity is registered monthly by the SIH/SUS based on the procedures recorded in the Hospital Admission Authorizations (*Autorizações de Internação Hospitalar*—AIHs) [9,13].

Both variables were expressed as person-year incidence (per 100,000 procedures or hospitalizations) to adjust for demographic variations over the past ten years, as well as to normalize them across territories (regions or states) with different population densities. Hence, population size estimates between 2014 and 2023 were retrieved from intercensal projections by the Brazilian Institute of Geography and Statistics (*Instituto Brasileiro de Geografia e Estatística*—IBGE). These projections do not provide robust estimates of the number of disabled persons in Brazil, and general population estimates were used instead [14].

As secondary variables, from the National Health Survey (2013, *Pesquisa Nacional de Saúde*—PNS) [15], general and oral health-related indicators were retrieved: (1) percentage of the estimated number of disabled persons (congenital or acquired disability); (2) proportion of inhabitants who self-rated their general health as “poor” or “very poor”; (3) proportion of inhabitants who self-rated their oral health as “poor” or “very poor”; (4) proportion of inhabitants who have never visited a dentist; (5) proportion of inhabitants who last visited a dentist for tooth extraction; (6) proportion of inhabitants who have lost 13 or more permanent teeth; (7) proportion of inhabitants who use some type of dental prosthesis. The first indicator mentioned was retrieved from Module G (indicators of disabled persons), and the remaining indicators were obtained from Module U (indicators of oral health) of the PNS questionnaire used in the 2013. In addition, from the Brazilian National Register of Healthcare Establishments (*Cadastro Nacional de Estabelecimento de Saúde*—CNES), the number of registered special care dentistry specialists in healthcare establishments in Brazil was retrieved [13].

### 2.3. Data Acquisition

The data acquisition was based on procedures previously described in all of the data sources [9,11]. The same researcher conducted the data collection, with prior experience in this procedure. For all variables, the TabNet tool was used to access the mentioned information systems and retrieve the data according to the study design. This tool is available on an internet-based virtual platform and enables immediate access to aggregated data, providing filters to adjust variables as needed [13].

In the SIA/SUS, the code #223288 (Special Care Dentistry/*Odontologia para Pacientes com Necessidades Especiais*) was used to filter the ambulatory productivity of these dental practitioners in Brazil’s public healthcare services. In the SIH/SUS, the code #0414020413 (Dental Care for Disabled Persons/*Tratamento Odontológico para Pacientes com Necessidades Especiais*). This code serves as an umbrella code for the provision of one or more dental procedures under general anesthesia or sedation in persons with one or more disabilities (permanent or temporary) of an intellectual, physical, sensory, and/or emotional nature that impedes them from undergoing conventional dental treatment [16].

Moreover, in both systems, the productivity approved by Brazil’s public healthcare system was considered. The territory and timeframe were adjusted according to the study design, and the other codes were applied in their respective filters, including adjustments for geographic scope. For the secondary variables, on the other hand, no specific code was needed, as the data were directly accessed in their original form, considering only the adjustments related to the territory and period designed in this study.

### 2.4. Data Analysis

After the data acquisition and exportation to spreadsheets, all datasets underwent processing and statistical analysis. The statistical packages JAMOVI (version 2.5.3, Sydney, Australia) and PAST (version 4.03, Oslo, Norway) were used, adjusting the significance level at 5% (α), considering all *p*-values less than 0.05 as a statistically significant outcome. Descriptively, in addition to the person-year incidence, the relationship between outpatient and inpatient dental care (procedures and hospitalizations) was also evaluated by the overall and annual average proportions, including its 95% confidence interval (CI) and all minimum and maximum values. Absolute (f) and relative (fr) frequencies were also provided [17].

Inferentially, comparisons between the person-year incidences of different territories (regions and national estimates) were conducted using generalized linear models (GLMs). Upon examining the outpatient and inpatient datasets, a Poisson distribution with robust variance was observed, indicating overdispersed count data. Consequently, a negative binomial regression analysis was applied to employ more flexible GLM adjustments. The maximum likelihood method was used to estimate all coefficients, considering the computation of the incidence ratio in the logarithmic link function (*Log*-likelihood). The reference level was the national estimate (Brazil) in all comparisons. Moreover, Spearman’s matrix was employed to examine correlation using the *rho* (ρ) coefficients [17].

The analysis of temporal trends over the past ten years was based on the use of Prais–Winsten regression analysis to estimate all angular coefficients (β_1_) while minimizing the effect of serial autocorrelation [9,18]. Thus, a logarithmic transformation (*log*10) of all dependent variables (incidences) was performed prior to estimation, including adjusted coefficients of determination (R^2^). The temporal variation, when significant, was expressed by annual percent change (APC), computed using the formula: APC (%) = [−1 + 10^(β1)^] × 100. The 95% CI for β_1_ (upper and lower limits) were computed using the formula: [β_1_ ± (*t*-critical value × β_1_-standard error)]. A non-significant *p*-value indicated stationarity. However, all analyses with an adjusted R^2^ equal to or higher than 20% (0.200) were redone to assess the possible impact of the COVID-19 pandemic years.

## 3. Results

Table 1 presents the frequency and person-year incidence (per 100,000 inhabitants) of outpatient dental procedures carried out by special care dentistry specialists (outpatient dental care) and hospitalizations for dental procedures of disabled persons (inpatient dental care) within Brazil’s public healthcare system from 2014 to 2023, both stratified by regions. It was observed that when disregarding the population factor (relative frequency), the southeastern and northeastern regions accounted for almost 70% of all outpatient dental procedures carried out (highest values), whereas the southern region showed the lowest value. However, when considering the population factor (incidence), the northern region showed the highest value (despite greater variability over time), and the south region remained with the lowest value.

Regarding inpatient dental care, relative frequency also demonstrates a predominance of the southern and southeastern regions in terms of productivity (highest values), comprising nearly 80% of all hospitalizations for dental treatment of disabled persons in Brazil’s public healthcare system over the last ten years, whereas the northern region showed the lowest value. However, when considering the incidence, the southeast and south regions showed the highest values, while the north region remained with the lowest values.

Table 2 presents the proportion and correlation of outpatient and inpatient dental care for disabled persons within Brazil’s public healthcare system from 2014 to 2023 in each region. It was observed that the north region had the highest proportion of outpatient dental procedures compared to hospitalizations, accompanied by the northeast, central–western, southeastern, and southern regions, respectively. However, the correlations indicate that solely the southern and central–western regions showed significantly similar (positive) temporal variations for both outpatient and inpatient dental care in this timeframe, with a very large effect size. The other regions showed distinct temporal variation patterns between the types of care (no significant correlations).

Table 3 presents the temporal trend of the person-year incidence of outpatient dental procedures carried out by special care dentistry specialists and hospitalizations for dental procedures of disabled persons within Brazil’s public healthcare system from 2014 to 2023, both stratified by regions. It was possible to observe that no region showed significant temporal variation in the last ten years. However, even with an insignificant *p*-value for this timeframe, four analyses presented R^2^ equal to or greater than 20% (0.200), in which the primary analysis was redone.

After removing the interval after the COVID-19 pandemic onset (evaluating from 2014 to 2019) in outpatient dental care, it was observed that there was an increasing trend in the incidence of outpatient dental procedures carried out by special care dentistry specialists in the southeastern region (*p*-value = 0.016, R^2^ = 0.846, APC (%) = 23.0%, 95% CI = 13.5–36.8%) and central–western region (*p*-value = 0.029, R^2^ = 0.833, APC (%) = 12.2%, 95% CI = 4.2–16.9%). Moreover, considering the inpatient dental care, it was observed that there was an increasing trend in hospitalizations for dental procedures of disabled persons in the south region (*p*-value = 0.002, R^2^ = 0.986, APC (%) = 14.3%, 95% CI = 13.0–16.4%). However, the north region maintained stationarity (*p*-value = 0.153). The effect size in this secondary analysis was very large for all regions reanalyzed.

Furthermore, Table 4 presents the comparisons of the person-year incidence (incidence ratio) of outpatient dental procedures carried out by Special Care Dentistry specialists and hospitalizations for dental procedures of disabled persons within Brazil’s public healthcare system from 2014 to 2023, considering the national estimate as the reference level. It was possible to observe that the disparities in the incidence values previously described were confirmed inferentially.

In outpatient dental care, the northern region showed a significantly higher person-year incidence than the national estimate, whereas the southern region was significantly below, and the northeastern, southeastern and central–western regions were similar (non-significant *p*-value). However, in inpatient dental care, the northern, northeastern, and central–western regions were significantly below the national estimate, whereas the southern region was significantly above, and the southeastern region was similar (non-significant *p*-value). The values of adjusted R^2^ for the models (outpatient and inpatient) were 60% and 67.9% (moderate level of explanation), respectively.

**Figure 1:**
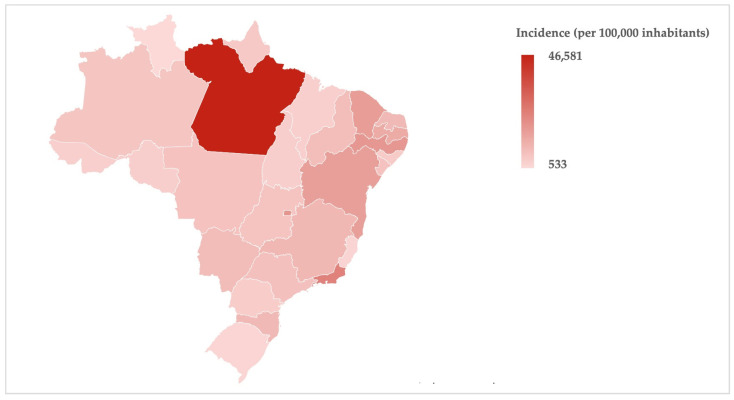
Person-year incidence of outpatient dental procedures carried out by special care dentistry specialists (outpatient dental care) within Brazil’s public healthcare system from 2014 to 2023.

**Figure 2:**
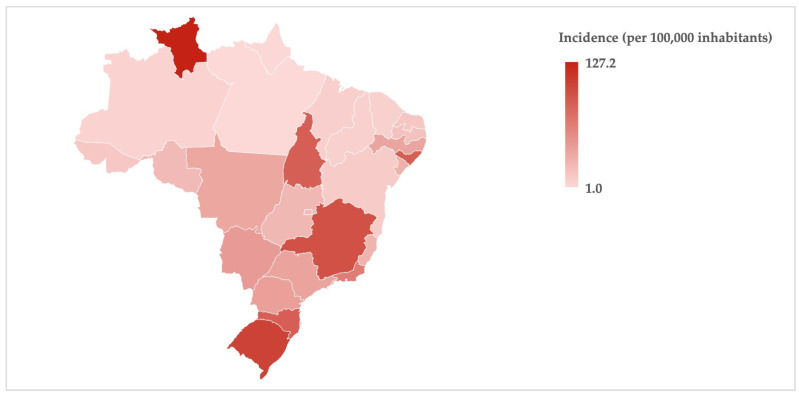
Person-year incidence of hospitalizations for dental procedures of disabled persons (inpatient dental care) within Brazil’s public healthcare system from 2014 to 2023.

It is possible to observe such differences on a map of Brazil by using a red color gradient to represent the person-year incidence of outpatient dental procedures carried out by special care dentistry specialists (Figure 1) and hospitalizations for dental procedures of disabled persons (Figure 2), both stratified by states (including the Federal District) and regions. The territorial disparities in dental care for disabled persons were pronounced among the regions compared to the national estimate, but it was also possible to observe intra-regional variabilities.

For instance, there was high outpatient productivity in the state of Pará (depicted in Figure 1 with the darker red color), located in the northern region, which presented a significantly higher incidence compared to the national estimate. However, it is possible to observe a lower incidence (lighter red color) in two neighboring states, Amapá and Roraima, as well as an intermediate red color in another neighboring state, Amazonas. Likewise, in Figure 2, it is also possible to observe that the three states that comprise the southern region (Rio Grande do Sul, Santa Catarina, and Paraná) showed three distinct color gradients (dark-to-light variation in red color), despite the highest inpatient productivity (incidence) occurring in this region.

Ultimately, Table 5 presents the correlation between the productivity of Brazil’s public healthcare system (outpatient and inpatient dental care for disabled persons) and oral health-related indicators. It was observed that there was a higher person-year incidence of outpatient dental procedures carried out by special care dentistry specialists in states with higher numbers of these dental practitioners in healthcare establishments, along with higher proportions of inhabitants who self-rated their general and oral health as “poor” or “very poor”, higher proportions of inhabitants who have never visited a dentist, and higher proportions of inhabitants whose last dental visit was for tooth extraction. Considering the sign and value of *rho* coefficients, all significant correlations in this analysis were positive and of low-to-moderate intensity. The other oral health-related indicators explored were not significantly correlated with outpatient dental care over the past ten years in Brazil’s public healthcare system. Furthermore, regarding inpatient dental care, there was no significant correlation between the person-year incidence of hospitalizations for dental procedures of disabled persons and the oral health-related indicators explored.

## 4. Discussion

This study evaluated territorial disparities in dental care for disabled persons in Brazil’s public healthcare system over the last ten years, exploring the correlation between productivity and oral health-related indicators through a population-level approach. The first null hypothesis was rejected, since territorial disparities in dental care for disabled persons were observed in outpatient and inpatient settings. The second null hypothesis was also rejected, since correlations between productivity and oral health-related indicators were observed.

In summary, the results presented indicate a predominance of outpatient dental care for disabled persons in all regions (safeguarding their respective proportions). However, except for the southern region, outpatient productivity did not correlate with inpatient productivity over time. This outcome is probably related to the oral healthcare model for disabled persons of Brazil’s public healthcare system: primary healthcare (PHC) serves as the reference level (first access), and special care dentistry specialists can provide the specialized care at dental specialty centers (*Centros de Especialidades Odontológicas*—CEOs), where it is a mandatory specialty (secondary healthcare—SHC). Both levels of dental care are outpatient-based [19,20]. It is important to highlight that, concerning the productivity of SHC, only 60% of DSCs offered the mandatory specialties in 2014 (the first year of this time series approach), in which special care dentistry is included. This could be related to the stationary trends observed over the past ten years [20].

Moreover, considering inpatient dental care, the milestones that strengthened this modality of dental care are relatively recent in Brazil’s healthcare system. It was only in 2014 that dental procedures in hospital settings were systematically recorded by SIH/SUS [17], and only in 2017 that an organized healthcare network for disabled persons was proposed, aiming to expand inpatient care for this population group (including dental care) [21,22]. In addition, it is worth noting that hospitalizations are often more costly and require specialized resources and professionals, which can limit the nationwide implementation of dental care for disabled persons in tertiary healthcare (THC) settings [9,22].

Nonetheless, only the southeastern region showed outpatient and inpatient productivity levels similar to the national estimate. The southern region showed divergent results, with low outpatient productivity and high inpatient productivity, whereas the north region showed the opposite pattern. The northeast and central–western regions were similar to the national estimate in outpatient productivity, but showed lower inpatient productivity. In addition, for the past ten years, it was observed that both outpatient and inpatient productivity did not show an increasing or decreasing trend, remaining stationary. However, it was demonstrated that this outcome was influenced by the COVID-19 pandemic in three out of the eight analyses.

The confirmation of these territorial disparities constitutes the main outcome of this study, providing a positive answer to the research question. It is worth noting that previous studies, also population-level approaches, have demonstrated how territorial disparities, including geographic location, can limit access to healthcare in a multidimensional point of view, which may be correlated with worse health outcomes and indicators [23,24], as was also demonstrated in this study regarding oral health-related indicators.

The concern in relation to the interface between OHRQoL and territorial disparities in dental care for disabled persons materializes, among other possibilities, with the outcomes of a previous study that demonstrated how weaknesses in access to PHC dental services can significantly impact OHRQoL. In this study, 412 Brazilian adults seeking dental care in Brazil’s public healthcare system were investigated through the Oral Health Impact Profile (OHIP-14) instrument. The participants responded to the question, “When your health center is open and you have an issue with your mouth or teeth, is there someone that can see you on the same day?” Those who answered negatively showed impacts on OHRQoL more frequently than those who answered positively [25].

In accessing specialized oral health services (SHC), despite the increase in the number of dental practitioners attending to disabled persons in DSCs, it was observed that 7.7% of these services still did not have this type of care in place in 2018, even though it was mandatory. Among the dental practitioners attending to disabled persons, only 37% were special care dentistry specialists. Moreover, 64% did not have protective stabilization, and 95.4% did not have sedation devices, as well as only 24.5% had a defined and agreed referral related to inpatient dental care (under general anesthesia or sedation) [26]. This evidence indicates that beyond the availability of service in each territory, the OHRQoL of disabled persons can be influenced by weaknesses specifically related to the resolving capacity of healthcare services, from the presence of specialists (as also demonstrated here) to the resources required for dental care [25,26]. The weaknesses in healthcare services mentioned support the perspective that there are disparities in dental care for this population, which may lead to unmet dental needs and worse oral health status [26], both directly linked to OHRQoL [25].

In relation to the COVID-19 pandemic, it was expected that it would affect the outcome of temporal trends, as sanitary restrictions to contain the spread of SARS-CoV-2 impacted the provision of dental care for disabled persons globally [27,28]. However, it is possible that this impact was greater on inpatient compared to outpatient dental care, as the COVID-19 scenario was often associated with hospitalizations, which may have more significantly limited the flow of disabled persons requiring dental care under general anesthesia or sedation [27]. The reduction in dental care was based on limitations in resources, dental practitioners available to attend to disabled persons, and the number of disabled persons seeking dental care during the lockdown period [25]. This scenario corroborates perspectives on the oral health of disabled persons in the post-COVID-19 era, hypothesizing a worsening of oral health status/OHRQoL during the pandemic period and an increased burden for dental care thereafter [28,29].

Addressing oral health-related outcomes through a population-level approach, it was demonstrated that outpatient productivity related to dental care for disabled persons was correlated with unfavorable oral health-related indicators relevant to OHRQoL, such as self-rated general and oral health, as well as higher proportions of inhabitants who had never visited a dentist or who visited for tooth extractions (last visit). This was an important outcome because it has been shown that poor self-rated oral health is a significant predictor of OHRQoL as assessed by valid instruments, and it is directly correlated with oral health practices/behaviors [30,31]. Therefore, a higher proportion of inhabitants who rate their oral health as “poor” or “very poor” is indicative that population-level OHRQoL may be below desirable levels.

Likewise, not visiting a dentist to address oral health issues has been associated with a higher propensity for oral diseases, such as dental caries [32]. In addition, it has also been demonstrated that there is association between tooth loss and OHRQoL, which is lower in individuals who have experienced more tooth loss over time [25]. Hence, it is important to emphasize that the assessment of these oral health-related indicators, which are relevant and commonly included in OHRQoL investigations, can be used to evaluate the impact of dental services on a population. This evaluation can guide the development of public policies and strategic actions to improve these indicators, consequently enhancing OHRQoL [1,2,33].

After this overview, it is crucial to report that differences in the incidence of procedures or hospitalizations among disabled persons may suggest two distinct outcomes across territories: (1) higher productivity could indicate poorer oral health conditions in that population, putting pressure on services (high demand for dental care), or (2) availability and access to Brazil’s public healthcare system dental services, as productivity can only be analyzed if such services exist. From this perspective, lower productivity may not reflect better oral health status, portraying the insufficient availability or absence of these services. The second perspective is supported by the positive correlation between productivity and the number of special care dentistry specialists, which is an oral health-related factor directly linked to service availability. However, considering that the territories with higher productivity also showed positive correlations with unfavorable oral health-related indicators, it is more likely that the first perspective gains strength in this setting, without neglecting the impact of service unavailability on this outcome.

In any case, the existence of territorial disparities cannot be understood positively, as it suggests that disabled persons living in different regions of Brazil face significant disparities in oral health status/OHRQoL, leading to varying demand-related productivities in Brazil’s public dental services, or they do not have equitable access to dental care through these services, either due to low resolution capacity or even unavailability in some territories, which would also contribute negatively to oral health status and OHRQoL.

Consequently, it is also necessary to incorporate the state of the art in understanding these results, particularly all evidence already mentioned about dental care for disabled persons in Brazil [9,19,20,21,22,26]. The territorial disparities highlighted in this study, coupled with the limitations of dental care for disabled persons in Brazil’s public healthcare system, allow us to take a step forward in understanding the worst oral health outcomes, including OHRQoL specific measures in future investigations. The combination of both perspectives (access-related barriers and challenges in providing dental care for disabled persons) can lead to poorer oral health-related indicators among the population in each territory, directly impacting the demand for dental care in the country (which justify the differences in productivity demonstrated here, whether at the outpatient or inpatient level).

Ultimately, it is important to note that this study had significant advantages, as it covered the entire national territory and a substantial timeframe, addressing both outpatient and inpatient dental care. However, it is not possible to apply all these outcomes and perspectives in the future without considering their limitations. The correlation between productivity and oral health-related indicators was observed at the population level, which restricts it to an ecological context and may not accurately reflect individual-level factors, potentially oversimplifying or hindering person-specific features. Furthermore, these indicators were related to the general population and not specifically to disabled persons. In addition, it is possible that other dental practitioners besides special care dentistry specialists provided dental care for disabled persons, which was not considered in this investigation. Thus, a new investigation could address OHRQoL using a specific instrument to understand access to dental health services from a territorial perspective, advancing the state of the art at an individual-level approach.

## 5. Conclusions

Territorial disparities were observed in outpatient and inpatient dental care for disabled persons in Brazil’s public healthcare system over the past ten years. Moreover, unfavorable oral health-related indicators were correlated with higher outpatient productivity for disabled persons. Therefore, these territorial disparities may contribute to oral health-related inequities among disabled persons. Hence, oral healthcare actions in this country, including OHRQoL investigations, should consider the territory where they live as an important component, addressing its related indicators and access to public dental services to better understand the outcomes relevant to disabled persons.

## Figures and Tables

**Table 1:** Frequency and person-year incidence of outpatient and inpatient dental care for disabled persons within Brazil’s public healthcare system from 2014 to 2023 (2024).

Territory (Region)	*f*	*fr* (%)	Incidence (100,000)	Annual Average	Minimum	Maximum
Outpatient dental care (procedures)						
Northern	4,383,764	19.4	23,946	2420 [896–3944]	579	7940
Northeastern	6,794,848	30.0	11,938	1196 [927–1465]	440	1721
Southeastern	8,877,289	39.2	10,083	1005 [791–1220]	515	1397
Southern	1,221,940	5.4	4092	409 [330–488]	176	528
Central-Western	1,352,037	6.0	8350	830 [636–1024]	439	1298
Inpatient dental care (hospitalizations)						
Northern	2878	3.2	15.7	1.58 [1.26–1.90]	0.79	2.21
Northeastern	11,113	12.2	19.5	1.95 [1.49–2.41]	0.93	2.75
Southeastern	49,610	54.5	56.3	5.61 [4.37–6.85]	2.98	9.00
Southern	22,631	24.9	75.8	7.56 [5.36–9.76]	4.11	13.8
Central–Western	4696	5.2	29.0	2.89 [2.31–3.47]	1.51	3.92

*f*: absolute frequency. *fr*: relative frequency. [ ]: 95% confidence interval.

**Table 2:** Proportion and correlation of outpatient and inpatient dental care for disabled persons within Brazil’s public healthcare system from 2014 to 2023 (2024).

Territory (Region)	Overall Proportion (O/H)	Annual Average Proportion (O/H)	Minimum	Maximum	ρ	*p*-Value
Northern	1523	1780 [507–3054]	295	5556	−0.309	0.387
Northeastern	611	650 [426–924]	372	1374	−0.067	0.865
Southeastern	179	192 [133–251]	109	384	0.418	0.232
Southern	54.0	56.9 [48.6–65.2]	38	71	0.964	<0.001 *
Central–Western	287	288 [256–320]	215	363	0.867	0.003 *

O: outpatient dental care (procedures). H: hospitalizations. [ ]: 95% confidence interval. ρ: Spearman’s *rho* coefficient. *: *p*-value < 0.05 (statistically significant outcome).

**Table 3:** Temporal trend of the person-year incidence of outpatient and inpatient dental care for disabled persons within Brazil’s public healthcare system from 2014 to 2023 (2024).

Territory (Region)	β_1_	R^2^	*p*-Value	Trend
**Outpatient dental care (procedures)**				
Northern	0.004 [−0.094, 0.074]	0.001	0.936	Stationary
Northeastern	−0.014 [−0.042, 0.023]	0.066	0.499	Stationary
Southeastern	0.031 [−0.001, 0.066]	0.402	0.061	Stationary
Southern	0.003 [−0.016, 0.043]	0.003	0.896	Stationary
Central–Western	0.026 [−0.008, 0.059]	0.248	0.140	Stationary
**Inpatient dental care (hospitalizations)**				
Northern	−0.023 [−0.040, 0.009]	0.201	0.189	Stationary
Northeastern	0.017 [−0.009, 0.052]	0.073	0.474	Stationary
Southeastern	0.018 [−0.003, 0.054]	0.120	0.327	Stationary
Southern	0.031 [−0.005, 0.072]	0.234	0.156	Stationary
Central–Western	0.058 [−0.011, 0.050]	0.048	0.542	Stationary

β_1_: angular coefficient. R^2^: coefficient of determination. [ ]: 95% confidence interval.

**Table 4:** Comparisons of the person-year incidence of outpatient and inpatient dental care for disabled persons within Brazil’s public healthcare system from 2014 to 2023 (2024).

Territory (Region)	Incidence Ratio	Limits		*z*-Score	*p*-Value
		Lower	Upper		
Outpatient dental care (procedures)					
Intercept	1011	915	1117		<0.001 *
Brazil	*ref*				
Northern	2.237	1.596	3.156	4.59	<0.001 *
Northeastern	1.105	0.783	1.560	0.57	0.568
Southeastern	0.930	0.659	1.312	−0.47	0.678
Southern	0.378	0.268	0.534	−5.52	<0.001 *
Central–West	0.768	0.544	1.084	−1.50	0.133
Inpatient dental care (hospitalizations)					
Intercept	2.88	2.44	3.40		<0.001 *
Brazil	*ref*				
Northern	0.308	0.161	0.588	−3.57	<0.001 *
Northeastern	0.359	0.195	0.661	−3.29	0.001 *
Southeastern	1.359	0.899	2.055	1.45	0.146
Southern	1.846	1.250	2.726	3.08	0.002 *
Central–Western	0.590	0.352	0.987	−2.01	0.045 *

*: *p*-value < 0.05 (statistically significant outcome). *ref*: reference level (incidence ratio = 1).

**Table 5:** The correlation between the productivity of Brazil’s public healthcare system (outpatient and inpatient dental care for disabled persons) and oral health-related indicators (2024).

Oral Health-Related Indicators	ρ	*p*-Value
Outpatient dental care (procedures)		
Estimate of the number of disabled persons (congenital or acquired disability)	N/A	0.439
Number of registered special care dentistry specialists in healthcare establishments	0.671	<0.001 *
Proportion of inhabitants who self-rated their general health as “poor” or “very poor”	0.440	0.025 *
Proportion of inhabitants who self-rated their oral health as “poor” or “very poor”	0.459	0.016 *
Proportion of inhabitants who have never visited a dentist	0.389	0.047 *
Proportion of inhabitants who last visited a dentist for tooth extraction	0.479	0.012 *
Proportion of inhabitants who have lost 13 or more permanent teeth	N/A	0.845
Proportion of inhabitants who use some type of dental prosthesis	N/A	0.744
Inpatient dental care (hospitalizations)		
Estimate of the number of disabled persons (congenital or acquired disability)	N/A	0.506
Number of registered special care dentistry specialists in healthcare establishments	N/A	0.269
Proportion of inhabitants who self-rated their general health as “poor” or “very poor”	N/A	0.726
Proportion of inhabitants who self-rated their oral health as “poor” or “very poor”	N/A	0.862
Proportion of inhabitants who have never visited a dentist	N/A	0.366
Proportion of inhabitants who last visited a dentist for tooth extraction	N/A	0.912
Proportion of inhabitants who have lost 13 or more permanent teeth	N/A	0.515
Proportion of inhabitants who use some type of dental prosthesis	N/A	0.080

ρ: Spearman’s *rho* coefficient. *: *p*-value < 0.05 (statistically significant outcome). N/A: not applicable (non-significant *p*-value).

## Data Availability

The data that support the finding of this study can be available on request from the corresponding author.

## References

[1] Yu X., Chen Y., Li Y., Hong J., Hua F. (2023). A bibliometric mapping study of the literature on oral health-related quality of life. J. Evid. Based Dent. Pract..

[2] Clementino L.C., de Souza K.S.C., Castelo-Branco M., Perazzo M.F., Ramos-Jorge M.L., Mattos F.F., Paiva S.M., Martins-Júnior P.A. (2022). Top 100 most-cited oral health-related quality of life papers: Bibliometric analysis. Community Dent. Oral Epidemiol..

[3] Yoo S.Y., Kim H.J., Kim S.K., Heo S.J., Koak J.Y., Park J.M. (2023). Quality of life in patients in South Korea requiring special care after fixed implants: A retrospective analysis. BMC Oral Health.

[4] Pradhan A., Keuskamp D., Brennan D. (2016). Oral health-related quality of life improves in employees with disabilities following a workplace dental intervention. Eval. Program Plann..

[5] Aguiar I., Lins-Kusterer L., Lins L.S., Paraná R., Bastos J., Carvalho F.M. (2019). Quality of life, work ability and oral health among patients with chronic liver diseases. Med. Oral Patol. Oral Cir. Bucal.

[6] Ohi T., Murakami T., Komiyama T., Miyoshi Y., Endo K., Hiratsuka T., Satoh M., Asayama K., Inoue R., Kikuya M. (2022). Oral health-related quality of life is associated with the prevalence and development of depressive symptoms in older Japanese individuals: The Ohasama Study. Gerodontology.

[7] Bjørkvik J., Henriquez-Quintero D.P., Vika M.E., Nielsen G.H., Virtanen J.I. (2022). Barriers and facilitators for dental care among patients with severe or long-term mental illness. Scand. J. Caring Sci..

[8] Peres M.A., Macpherson L.M.D., Weyant R.J., Daly B., Venturelli R., Mathur M.R., Listl S., Celeste R.K., Guarnizo-Herreño C.C., Kearns C. (2019). Oral diseases: A global public health challenge. Lancet.

[9] Lima R.B., Vilela L.D., Nelson-Filho P., da Silva L.A.B., da Silva R.A.B. (2023). Caries-related hospital morbidity in the Brazilian Unified Health System from 2008 to 2022. Braz. Oral Res..

[10] Thomaz E.B.A.F., Costa E.M., Queiroz R.C.S., Emmi D.T., Ribeiro A.G.A., Silva N.C.D., Hugo F.N., Figueiredo N. (2022). Advances and weaknesses of the work process of the oral cancer care network in Brazil: A latent class transition analysis. Community Dent. Oral Epidemiol..

[11] Lima R.B., de Barros M.L.T., Gomes e Moura A.P., Nelson Filho P., da Silva R.A.B., da Silva L.A.B. (2023). Endodontic treatment in the Unified Health System in the North and Southeast regions of Brazil: 15-year evaluation. SaudColetiv.

[12] Resolution 510 of April 7, 2016-Brazil, National Health Council-Ministry of Health. *Dispõe Sobre as Normas Aplicáveis a Pesquisas em Ciências Humanas e Sociais*. https://conselho.saude.gov.br/.

[13] Department of Informatics (DATASUS)-Brazil, National Health Council-Ministry of Health Acesso à Informação.

[14] Brazilian Institute of Geography and Statistics (IBGE)-Brazil, Ministry of Planning and Budget Estatísticas.

[15] National Health Research (PNS)-Brazil, Ministry of Health Painel de Indicadores de Saúde.

[16] Department of Informatics (DATASUS)-Brazil, National Health Council-Ministry of Health Tabela de Procedimentos, Medicamentos, Órteses, Próteses e Materiais Especiais.

[17] Pagano M., Gauvreau K., Heather M. (2022). Principles of Biostatistics.

[18] Antunes J.L., Cardoso M.R. (2015). Using time series analysis in epidemiological studies. Epidemiol. Serv. Saude.

[19] Stein C., Santos K.W.D., Condessa A.M., Celeste R.K., Hilgert J.B., Hugo F.N. (2019). Presence of Specialized Dentistry Centers and the relationship with dental extractions in the oral healthcare network in Brazil. Cad. Saude Publica.

[20] Rios L.R.F., Colussi C.F. (2019). Analysis of the supply of specialized oral health care services in the Brazilian National Health System: Brazil, 2014. Epidemiol. Serv. Saude.

[21] Carneiro J.D.B., Bousquat A.E.M., Frazão P. (2022). A Implementation of the care network for people with disabilities in the scope of oral health from the advocacy coalition framework in two health regions in Brazil. Adm. Pub. Gest. Soc..

[22] Carvalho L.F., Leite I.C.G., Farah B.F. (2023). Oral health care network for people with disabilities: Challenges and potentialities of Primary Health Care. Res. Soc. Dev..

[23] Zon H., Pavlova M., Groot W. (2020). Regional health disparities in Burkina Faso during the period of health care decentralization. Results of a macro-level analysis. Int. J. Health Plann. Manag..

[24] Zon H., Pavlova M., Groot W. (2021). Factors associated with access to healthcare in Burkina Faso: Evidence from a national household survey. BMC Health Serv. Res..

[25] Bastos L.F., Hugo F.N., Hilgert J.B., Cardozo D.D., Bulgarelli A.F., Santos C.M.D. (2019). Access to dental services and oral health-related quality of life in the context of Primary Health Care. Braz. Oral Res..

[26] Queiroz R.C.S., Oliveira I.C.V., Silva N.C.D., Borges T.S., Nunes A.M.M., Figueiredo N., Thomaz E.B.A.F. (2022). Oral health care for people with disabilities in Brazil: Transition from the specialized dental services between 2014 and 2018. Community Dent. Oral Epidemiol..

[27] Tewfik K., Peta C., De Giuli M.C., Rossini M., Giampaoli G., Covelli C., Burlini D. (2023). Impact of COVID-19 pandemic on children with special needs requiring general anaesthesia for the treatment of dental disease: The experience of the Brescia Children’s Hospital, Lombardy, Italy. Eur. Arch. Paediatr. Dent..

[28] Phadraig C.M.G., van Harten M.T., Diniz-Freitas M., Posse J.L., Faulks D., Dougall A., Dios P.D., Daly B. (2021). The impact of COVID-19 on access to dental care for people with disabilities: A global survey during the COVID-19 first wave lockdown. Med. Oral Patol. Oral Cir. Bucal..

[29] Dziedzic A., Tanasiewicz M., Tysiąc-Miśta M. (2020). Dental are provision during coronavirus disease 2019 (COVID-19) pandemic: The importance of continuous support for vulnerable patients. Medicina.

[30] Zheng S., Zhao L., Ju N., Hua T., Zhang S., Liao S. (2021). Relationship between oral health-related knowledge, attitudes, practice, self-rated oral health and oral health-related quality of life among Chinese college students: A structural equation modeling approach. BMC Oral Health.

[31] Yin N., Li W., Zhou H., Zhang Y., Zhang W., Ding W., Ge H., Zhang S., Liao S. (2023). Associations among self-rated oral health, subjective oral conditions, oral health behaviours, and oral health-related quality of life (OHRQoL). Oral Health Prev. Dent..

[32] Singla N., Acharya S., Singla R., Nayak P. (2020). The impact of lifestyles on dental caries of adult patients in Udupi district: A cross-sectional study. J. Int. Soc. Prev. Community Dent..

[33] Spanemberg J.C., Cardoso J.A., Slob E.M.G.B., López-López J. (2019). Quality of life related to oral health and its impact in adults. J. Stomatol. Oral Maxillofac. Surg..

